# Accelerometer-Measured Physical Activity at Work and Need for Recovery: A Compositional Analysis of Cross-sectional Data

**DOI:** 10.1093/annweh/wxz095

**Published:** 2019-12-27

**Authors:** Matthew L Stevens, Patrick Crowley, Charlotte L Rasmussen, David M Hallman, Ole S Mortensen, Clas-Håkan Nygård, Andreas Holtermann

**Affiliations:** 1 Musculoskeletal Disorders and Physical Workload, The National Research Centre for the Working Environment, Copenhagen, Denmark; 2 Section of Social Medicine, Department of Public Health, University of Copenhagen, Copenhagen K, Denmark; 3 Department of Occupational Health Sciences and Psychology, Centre for Musculoskeletal Research, University of Gävle, Gävle, Sweden; 4 Department of Occupational and Social Medicine, Copenhagen University Hospital Holbæk, Holbæk, Denmark; 5 Unit of Health Sciences, Faculty of Social Science, Tampere University, Tampere, Finland; 6 Department of Sports Science and Clinical Biomechanics, University of Southern Denmark, Odense M, Denmark

**Keywords:** blue-collar workers, compositional data analysis, physical activity, physical behaviour, need for recovery, triaxial accelerometers

## Abstract

**Objectives:**

Previous research has shown strong associations between occupational physical activity (OPA) and need for recovery (NFR). However this research has only utilized self-reported measures of OPA which may be biased. Thus, there is a need for investigating if the previously documented association between self-reported OPA and NFR can be found when using technical measures of OPA. There is also the need to investigate whether older workers are particularly susceptible to increased NFR, since age-related declines in physical capacity mean that it is likely these workers will have a higher NFR for a given physical activity. The aim of this study was to investigate the association between technically measured OPA and NFR, and whether this relationship is modified by age.

**Methods:**

This study utilized data from the Danish Physical Activity Cohort with Objective Measurements cohort—comprising Danish workers (*n* = 840) from the cleaning, manufacturing, and transportation sectors. OPA was measured by accelerometers attached to the thigh and upper back for at least one work day and classified into four physical behaviour categories (sedentary, standing, light, or moderate/vigorous). NFR was measured using a shortened version of the Danish NFR scale. Analysis was conducted using linear regression and isotemporal substitution analyses for compositional data.

**Results:**

The overall association between OPA and NFR was statistically significant in the unadjusted model (*P* < 0.001), but not when adjusted for age, sex, occupation, and shift work (*P* = 0.166). Isotemporal substitution showed small but significant reductions in NFR when increasing sedentary time relative to other behaviours (adjusted: ΔNFR = −0.010 [−0.019; −0.001]). There were no significant interactions between age and OPA (*P* = 0.409).

**Conclusions:**

This study found significant associations between OPA and NFR, but the effect sizes were small. Reallocating 30 min to sedentary behaviours from other behaviours was associated with a reduced NFR, but the effect size may not be practically relevant. Moreover, no clear modifying effects of age were identified.

## Introduction

The ageing population is a major challenge for modern economies. This is mainly because increased age will increase the proportion of the population outside the workforce relative to those being productively employed (OECD, 2006). To combat this relative decline in the proportion of the population productively employed, governments all over the world are increasing the statutory retirement age (OECD, 2017). However, increasing the retirement age comes with several challenges, particularly for vulnerable groups (e.g. low skilled, manual workers) (Andersen *et al.*, 2016; Kadefors *et al.*, 2019). This is because simply increasing the retirement age does not change the capacity of workers to conduct their work (Schofield *et al.*, 2008; Kadefors *et al.*, 2019). Thus, to enable these vulnerable groups to remain in the workforce until the elevated retirement age, a thorough understanding of the risk factors for leaving the workforce is required.

A key predictor of a person’s likelihood of leaving the workforce is their ‘need for recovery’ (NFR) (van Veldhoven and Broersen, 2003; Stynen *et al.*, 2019). NFR was first conceptualized as an outcome measure in 1994 (Van Veldhoven and Meijman, 1994) and measures the interaction between a worker’s physical and mental workload, their capacity to work and their capacity to continuously recover from that workload. NFR has shown strong associations with related measures such as fatigue and emotional exhaustion (van Veldhoven and Broersen, 2003), as well as work absenteeism in multiple workgroups (van Veldhoven, 1996; De Croon *et al.*, 2003) and thus is a useful intermediate outcome measure in occupational health research. Therefore, knowledge about the factors predicting increased NFR is important for early prevention of work-related symptoms, work absenteeism, and early retirement.

One factor that has shown to be associated with increased NFR is high occupational physical activity (OPA) (Kraaijeveld *et al.*, 2014; Gommans *et al.*, 2016). However, our understanding of this relationship is hindered by important limitations in how existing studies have measured and analysed OPA. Firstly, the existing studies have utilized self-reported measures of OPA, which show limited accuracy (Steene-Johannessen *et al.*, 2016). Furthermore, such analyses have not considered the time dependency of physical behaviours like OPA—meaning that if more time is spent in one behaviour then less time is available to be spent in other behaviours. This has repercussions for the interpretation of results, since any effect on a health outcome is therefore a result of the trade-off between time spent in two or more behaviours rather than only time spent in a single isolated behaviour. Thus, to correctly understand the health effects of OPA, analytical approaches that consider the proportion of time spent engaging in different behaviours (e.g. sitting, standing, walking, and running) are required.

To address the limitations of previous research investigating the relationship between OPA and NFR, novel solutions are required. The first is to use technical measures of OPA to increase the validity of the physical behaviours identified. These measures can be obtained from accelerometers attached to the thigh and upper back and demonstrate high accuracy for identifying physical behaviours (Skotte *et al.*, 2014; Stemland *et al.*, 2015). The second is to use compositional data analyses (CoDA) to address the codependency of time spent in different behaviours (Aitchison, 1982; Pedišić *et al.*, 2017). Using CoDA not only facilitates investigation of effects of time in different behaviours relative to the others but also allows for compositional isotemporal substitution modelling (Dumuid *et al.*, 2019), which estimates the effects of reallocating time to/from different behaviours.

Another factor that has been associated with NFR is age, with several studies showing that increased age is associated with an increased NFR (Kiss *et al.*, 2008; Verdonk *et al.*, 2010; Kraaijeveld *et al.*, 2014). The proposed explanation for this is that age-related declines in physical capacity will affect the ability of individuals to perform their work-tasks. Older workers will therefore be expected to work closer to their maximal capacity, and will subsequently have increased NFR (Kiss *et al.*, 2008; Verdonk *et al.*, 2010). As such, it can be expected that the effect of OPA on NFR is more pronounced in older workers compared to younger workers. However, only a few studies have previously investigated if older workers have a higher NFR when performing high OPA than younger workers (Sluiter, 2003; Kiss *et al.*, 2008; Verdonk *et al.*, 2010), and no study has investigated this using technical measurements of OPA.

The aim of this study was to investigate the association between technically measured OPA and NFR, and if age modifies the association between OPA and NFR.

## Methods

This was an analysis of cross-sectional, baseline data from a large prospective cohort study—the Danish Physical Activity Cohort with Objective Measurements (DPhacto) (Jørgensen *et al.*, 2013). The aim of DPhacto was to investigate the association between accelerometer-measured physical activities at work and health amongst blue-collar workers. The DPhacto study was approved by the Danish data protection agency and local Ethics Committee (H-2-2012-011). Full details of this cohort have been provided in the previously published protocol (Jørgensen *et al.*, 2013) and cohort profile (Jørgensen *et al.*, 2019). As such, only details of the methods relevant to this analysis have been provided below.

### Participants

Participants were recruited from 15 workplaces in three different sectors (cleaning, manufacturing, and transportation). All blue-collar workers from these workplaces, as well as some white collar colleagues within the same workplaces, were invited to participate in the study through local information meetings.

### Data collection and outcomes

Data for this analysis came from the DPhacto baseline data collection which included questionnaires, health checks, and accelerometer-based physical activity measurements. All eligible workers were invited to complete the questionnaire and to participate in the health check, which consisted of anthropometric measurements and a physical health examination. Participants were asked to wear accelerometers for a minimum of two consecutive workdays and to complete a diary reporting time at work and non-wear time.

### Physical activity

Physical activity at work was assessed using data from two triaxial ActiGraph GT3X+ accelerometers (Actigraph, Pensacola, FL, USA). The accelerometers were fixed to the upper back and right thigh using double-sided adhesive tape (3M, Hair-Set, St. Paul, MN, USA) and Fixomull (Fixomull BSN medical GmbH, Hamburg, Germany). Accelerometer data were downloaded using Actilife Software version 5.5 (Actigraph, Pensacola, FL, USA) and the custom-made MATLAB program Acti4 (The National Research Centre for the Working Environment, Copenhagen, Denmark) (Skotte *et al.*, 2014) was used to determine the time spent in various physical behaviours (i.e. cycling, stair climbing, running, walking, standing, sitting, and lying). The Acti4 program has been shown to classify physical behaviours with high sensitivity and specificity under semistandardized (Skotte *et al.*, 2014) and free-living conditions (Stemland *et al.*, 2015). The method for classification of physical behaviours using Acti4 has been previously described (Skotte *et al.*, 2014).

Daily work hours were defined from the participants’ self-reported diary information. To be included in the analysis, workers had to have at least 1 day of valid accelerometer measurements at work. A valid day consisted of ≥4 h of accelerometer-derived work time or ≥75% of the individual’s daily average work time. For workers with more than one valid day of accelerometer measurements, the average daily time spent in OPA was calculated. For conducting the analysis, the physical behaviours were grouped into five classifications. These were non-work time, and time at work in: sedentary behaviours (lying and sitting), standing, light physical behaviours (dynamic standing and slow walking—defined as a cadence of less than 100 steps per min), and moderate/vigorous physical behaviours (fast walking—defined as a cadence of more than 100 steps per min, running, stair climbing, and cycling).

### Need for recovery

NFR was assessed using a validated short-form version of the Danish NFR scale. This version consisted of three items (‘I find it hard to relax *after* a working day,’ ‘At the end of my work day, I am exhausted,’ and ‘After a workday, I am too tired to begin other activities’) scored on a 5-point Likert scale with the response categories: ‘Never’; ‘Rarely’; ‘Some of the time’; ‘Most of the time’; and ‘Always’. For the analysis, a composite score was developed by taking the mean of the three items. This shortened version of the Danish NFR scale has shown excellent criterion validity (Intraclass Correlation Coefficient = 0.83–0.86) when compared to the full scale (Stevens *et al.*, 2019).

### Covariates/demographics

Demographic information including age, sex, body mass index (BMI), occupation, and shift work was also collected at baseline. BMI was calculated from height and weight measured at the health check and used as a continuous variable in all analyses. Occupation was categorized into four groups based upon the individual’s job sector (cleaning/manufacturing/transportation) and job type (administration/blue-collar). Due to expected similarities in physical activity at work for all workers classified as administrative, they were assigned to a single group, whilst blue-collar workers were split into three other groups according to their sector. Information on shift work was assessed using the question: ‘At what time(s) of the day do you usually work in your main occupation?’ with three response categories: fixed day work; night/varying work hours with night; and other. Years in position was assessed with the question ‘For how long have you had the kind of occupation as you have now?’ Perceived health was assessed using the question ‘How do you rate your overall health?’ on a 5-point Likert scale with responses from very poor to very good. Prescription medication was assessed using the question ‘Have you, in the last three months, taken prescription medication?’ (yes/no). Work ability was assessed with the question ‘Please rate your present work ability?’ on a 0–10 visual analogue scale (0 = not able to work, 10 = best ability to work).

### Statistical analyses

Analyses were conducted utilizing CoDA (Aitchison, 1982; Pedišić *et al.*, 2017; Dumuid *et al.*, 2018). This first involved expressing the relative time spent in the five behaviour classifications (non-work time, and time at work in sedentary behaviours, standing, light physical behaviours, and moderate/vigorous physical behaviours) using an isometric log-ratio (ilr)-coordinate system. This ilr-coordinate system consisted of four coordinates, which between them contained all information about the relative importance of each behaviour classification with respect to all other classifications. This conversion of the compositional data into an ilr-coordinate system allows for the data to be handled using standard statistical methods (e.g. regression analysis) (Hron *et al.*, 2012, 2017). Non-work time was included in the analysis to account for the total length of time spent at work.

In this study, the first ilr-coordinate contained the relative information between time at work and non-work time. The second ilr-coordinate contained the relative information between sedentary behaviours at work and the other behaviours (standing, light, and moderate/vigorous behaviours) at work. The third ilr-coordinate contained the relative information between standing at work and the remaining behaviours (light and moderate/vigorous behaviours) at work. Finally, the fourth ilr-coordinate contained the relative information between light and moderate/vigorous behaviours at work. The equations used to calculate these ilrs are provided in Appendix 1, available at *Annals of Work Exposures and Health* online.

Unfortunately, only the first ilr developed has interpretability since it expresses the total variance in the composition. The remaining ilrs (i.e. ilrs 2–4 in this study) lack interpretability because they only express part of the variance of, and are dependent upon, all preceding ilrs (Aitchison, 1982; Pedišić *et al.*, 2017; Dumuid *et al.*, 2018). Ilr 1 was chosen as the ratio of work to non-work time as (although not the aim of this study) an understanding of the relationship between work and non-work time provides complementary information to our aim of investigating the relationship different behaviours at work have with NFR. The use of ilrs to directly understand the relationship between physical behaviours and NFR was not possible because any ilr developed would also include non-work time. Additionally, because CoDA uses these ratios between behaviours as the exposure in the developed regression model, it is a requirement for these analyses that none of the behaviours themselves are zero. However, there were no zeros present in the composition analysed.

After constructing the ilr-coordinates, the analyses were conducted using linear regression modelling. First, an unadjusted model was developed that consisted of only the physical behaviour composition (four ilr-coordinates) as continuous predictors and NFR as a continuous dependent variable. The second model was an adjusted model that also included age, sex, occupation, and shift work. These potential confounders were chosen based on theoretical assumptions concerning their possible influence on physical behaviours at work and NFR. All potential confounders were kept in the adjusted model regardless of significance. Due to expected differences in the relationship between physical behaviours at work and NFR amongst those of different ages, analyses assessing the interaction with age and stratified by age were also prespecified. When including age in the model it was entered as a continuous variable. However, stratification by age was based upon tertiles of age distribution. Linearity assumptions were checked visually through the use of scatterplots (and LOWESS lines) assessing the developed ilrs against NFR and plots of the residuals versus predicted values.

Since the ilrs developed (by themselves) lack interpretability, isotemporal substitution modelling was used to provide effect estimates for the differences across the sample when reallocating time between physical behaviours on NFR in both the unadjusted and adjusted model (Dumuid *et al.*, 2019). This was done in two ways. Firstly, a one-to-many analysis was performed (Dumuid *et al.*, 2019). This entailed reallocating time spent in a single behaviour classification at work to/from all other behaviour classifications at work. This reallocation is done to retain the relative relationships between the other behaviour classifications (e.g. when reallocating time to two behaviour classifications, if behaviour classification one was performed twice as much as behaviour classification two it would receive twice as much of the reallocated time). Secondly, a one-to-one analysis was performed that reallocated time to/from moderate/vigorous physical behaviours at work from/to each other behaviour (i.e. sedentary behaviours, standing, and light physical behaviours) at work (Dumuid *et al.*, 2019).

The two different substitution methods were used because they provide different insights into what potential effects reallocating behaviour might have. The one-to-many analysis holds constant the ratios between all behaviours except those involving one particular behaviour; and thus provides a more theoretical understanding about the effects of changing that single behaviour. Conversely, the one-to-one analysis provides a more practical approach to understanding behaviour change from one to another. This simulates a situation where time spent at work in one type of behaviour, is now replaced by a different behaviour.

Non-work time was not included in any reallocation (i.e. we did not assess the effect of changing the amount of time spent at work). The significance of the differences between age groups on the estimated effect of reallocating time in the one-to-many and one-to-one analyses was evaluated using Welch modified two-sample *t*-tests (Arnholt and Evans, 2017). Confidence intervals for the substitution modelling were developed from the standard deviations computed in the regression model, which are applicable for use in the substitution model after centring the ilrs at the mean.

All analyses were conducted in RStudio v1.1.456 (RStudio Team, 2016)/R v3.5.1 (R Core Team, 2018) utilizing packages ‘compositions’ v1.40-2 (van den Boogaart *et al.*, 2018), ‘robcompositions’ v2.0.10 (Matthias *et al.*, 2019), ‘car’ v3.0-2 (Fox, 2018), ‘BSDA’ v1.2.0 (Arnholt and Evans, 2017), ‘lmtest’ v0.9-36 (Hothorn *et al.*, 2018), and ‘ggplot2’ v3.1.0 (Wickham *et al.*, 2018). All significance testing was based upon an *α* of 0.05. Because of the differences in the behaviours performed between each occupational group (admin, cleaning, manufacturing, and transportation), it was decided to conduct a sensitivity analysis that stratified by occupation in the adjusted model.

## Results

Of the 1087 participants providing data for DPhacto, 840 had data fulfilling the requirements of the current analysis. Of the 247 participants not contributing to this analysis, 243 did not provide adequate accelerometry data whilst a further 4 did not provide a response for NFR at baseline. Participants in this study were mostly middle aged (mean [SD] = 45.1 [9.8]) and just over half (54.3%) were male (Table 1). The majority (59.2%) of participants were blue-collar manufacturing workers and most (82.5%) had fixed day-time work. Nearly all (98.1%) perceived their health to be at least ‘fairly good’. The geometric mean (arithmetic mean) for time spent at work was approximately 6.5 (7.5) h, within which the mean sedentary time was roughly 2.5 (3) h, standing time was 2 (2.5) h, and time spent in both light physical behaviours and moderate/vigorous physical behaviours was 1 (1) h (Table 2).

**Table 1.** Participant demographics of workers from cleaning, manufacturing, and transportation sectors in Denmark.

	Mean (SD), median (IQR) or *n* (%)			
	Overall	Age stratified		
		≤40	41–50	≥51
Sex (m)	(*n* = 840)	(*n* = 259)	(*n* = 306)	(*n* = 275)
	456 (54.3%)	158 (61%)	154 (50.3%)	144 (52.4%)
Age	(*n* = 840)	(*n* = 259)	(*n* = 306)	(*n* = 275)
	45.1 (SD 9.8)	33.3 (SD 7.7)	45.9 (SD 2.7)	55.4 (SD 3.6)
Sector/occupation^a^	(*n* = 752)	(*n* = 232)	(*n* = 276)	(*n* = 244)
Administration	181 (24.1%)	49 (21.1%)	73 (26.4%)	59 (24.2%)
Cleaning	79 (10.5%)	22 (9.5%)	27 (9.8%)	30 (12.3%)
Manufacturing	445 (59.2%)	142 (61.2%)	163 (59.1%)	140 (57.4%)
Transport	47 (6.3%)	19 (8.2%)	13 (4.7%)	15 (6.1%)
Shift work	(*n* = 818)	(*n* = 255)	(*n* = 297)	(*n* = 266)
Fixed day work	675 (82.5%)	205 (80.4%)	245 (83.5%)	225 (84.6%)
Night/varying	100 (12.2%)	37 (14.5%)	35 (11.8%)	28 (10.5%)
Other	43 (5.3%)	13 (5.1%)	17 (5.7%)	13 (4.9%)
Years in position	(*n* = 808)	(*n* = 252)	(*n* = 293)	(*n* = 263)
	11 (IQR 5–20)	7 (IQR 4–12)	12 (IQR 5–21)	17 (IQR 8–28)
Smoking	(*n* = 820)	(*n* = 255)	(*n* = 299)	(*n* = 266)
Never	345 (42.1%)	116 (46.0%)	125 (41.8%)	104 (39.1%)
Former	249 (30.4%)	58 (22.7%)	89 (29.8%)	102 (38.3%)
Current	226 (27.6%)	81 (31.8%)	85 (28.4%)	60 (22.6%)
Perceived health	(n = 823)	(*n* = 257)	(*n* = 299)	(*n* = 267)
Very good	64 (7.8%)	22 (8.6%)	27 (9.0%)	15 (5.6%)
Good	512 (62.2%)	158 (61.5%)	193 (64.5%)	161 (60.3%)
Fairly good	231 (28.1%)	74 (29.0%)	72 (24.1%)	85 (31.8%)
Poor	15 (1.8%)	3 (1.2%)	7 (2.3%)	5 (1.9%)
Very poor	1 (0.1%)	0 (0.0%)	0 (0.0%)	1 (0.4%)
Prescription medication (y)	(*n* = 840)	(*n* = 259)	(*n* = 306)	(*n* = 275)
	338 (40.2%)	76 (29.6%)	118 (38.6%)	144 (52.4%)
BMI	(*n* = 822)	(*n* = 252)	(*n* = 301)	(*n* = 269)
	27.3 (SD 4.8)	26.9 (SD 5.1)	27.6 (SD 4.7)	27.4 (SD 4.5)
Work ability^b^	(*n* = 840)	(*n* = 259)	(*n* = 306)	(*n* = 275)
	9 (IQR 8–9)	9 (IQR 8–9)	9 (IQR 8–9)	9 (IQR 8–9)
NFR^c^	(*n* = 840)	(*n* = 259)	(*n* = 306)	(*n* = 275)
	2.5 (SD 0.7)	2.5 (SD 0.7)	2.5 (SD 0.7)	2.5 (SD 0.7)

SD = standard deviation; IQR = interquartile range.

^*a*^Workers classified as administration were drawn from all sectors (cleaning/manufacturing/transportation), leaving blue-collar workers classified according to their sector.

^*b*^Work ability was measured on a 0–10 numerical rating scale.

^*c*^NFR was measured on a 5-point Likert scale (higher scores indicate a higher NFR).

**Table 2.** Physical behaviour demographics described as the geometric mean and a variation matrix of workers from cleaning, manufacturing, and transportation sectors in Denmark (*n* = 840).

	SB	Standing	LPB	MVPB	Non-work
Geometric mean (min day^−1^)	143	128	67	57	1045
Variation matrix					
SB	0.00				
Standing	1.75	0.00			
LPB	2.80	0.37	0.00		
MVPB	1.60	0.36	0.33	0.00	
Non-work	0.94	0.31	0.53	0.32	0.00

The variation matrix shows the variance between the specified elements of the composition. The larger the number the greater the variance between the specified elements.

SB = sedentary behaviours (lying and sitting); LPB = light physical behaviours (dynamic standing and slow walking); MVPB = moderate/vigorous physical behaviours (fast walking, running, stair climbing, and cycling); non-work = time spent outside work (i.e. leisure, sleep, and transportation) on a work day.

When inspecting the geometric mean for time spent in various behaviours between ages there was a trend of decreased sedentary time at work amongst older workers with those aged 51 or older spending on average 22 min day^−1^ less in sedentary behaviours than those aged 40 or younger (Table 3). Comparison of the geometric means for behaviours across occupational groups showed that cleaners are the most active at work with the least time spent in sedentary behaviours (84 min day^−1^) and most time spent in both light (120 min day^−1^) and moderate/vigorous (80 min day^−1^) physical behaviours. Conversely the administration group was the least active with an average of 257 min day^−1^ spent sedentary, and only 32 and 38 min day^−1^ spent in light and moderate/vigorous behaviours, respectively. Full details are presented in Appendix 3, available at *Annals of Work Exposures and Health* online.

**Table 3.** Mean time spent in physical behaviour types at work for workers from cleaning, manufacturing, and transportation sectors in Denmark—stratified by age.

Age group	Geometric mean (min day^−1^)				
	SB	Standing	LPB	MVPB	Non-work
≤40 (*n* = 231)	153	126	61	56	1044
41–50 (*n* = 273)	149	131	65	56	1039
≥51 (*n* = 243)	131	134	72	55	1048

SB = sedentary behaviours (lying and sitting); LPB = light physical behaviours (dynamic standing and slow walking); MVPB = moderate/vigorous physical behaviours (fast walking, running, stair climbing, and cycling); non-work = time spent outside work (i.e. leisure, sleep, and transportation) on a work day.

### Physical behaviour at work on NFR

In the unadjusted model, there was a statistically significant association between the overall physical behaviour composition and NFR (*P* < 0.001). However, the adjusted model showed no significant association between the overall physical behaviour composition and NFR (*P* = 0.166). There was also no statistically significant association between age and NFR (*β* = 0.00; *P* = 0.422) nor statistically significant interaction between age and overall physical behaviour on NFR (*P* = 0.409). Moreover, sex was the only significant confounder in the adjusted model (*P* < 0.001). When considering the ilr_1_, there were no significant associations when increasing work time compared to non-work time (*β* = −0.12; *P* = 0.214 and *β* = −0.05; *P* = 0.615 in the unadjusted and adjusted models, respectively).

When the adjusted model was stratified by age (retaining gender, occupation, and shift work as potential confounders), no statistically significant associations between the overall physical behaviour compositions and NFR were identified (*P* > 0.05) and there were no significant associations between work time relative to non-work time and NFR (*P* > 0.05). Results for all ilrs are provided in Appendix 1, available at *Annals of Work Exposures and Health* online. In those aged 40 years and younger, both sex and occupation were significant confounders (*P* = 0.003 and 0.041, respectively). In those aged 41–50 years, sex and shift work were again significantly associated with NFR (*P* < 0.001 and *P* = 0.013, respectively). In those aged above 50 years none of the confounders were significantly associated with NFR (*P* > 0.05).

### Compositional isotemporal substitution modelling

**Figure 1.**
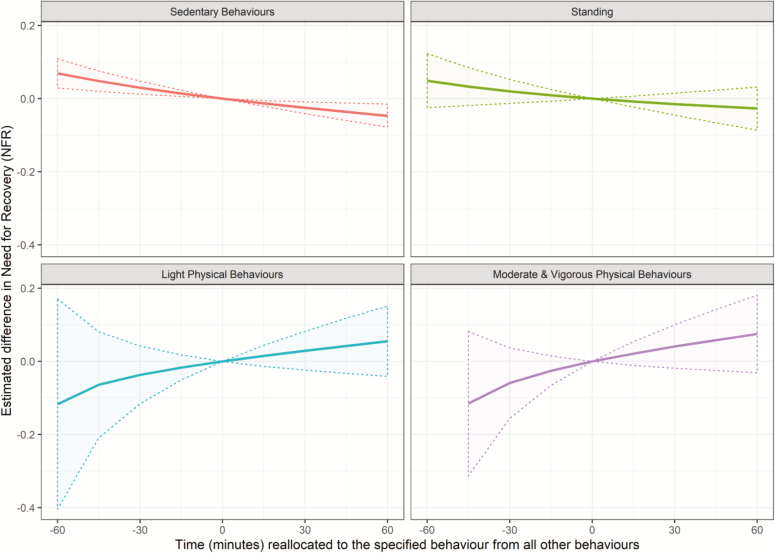
Estimated difference (±95% CI) in NFR (measured on a 5-point Likert scale) when reallocating time to a specific behaviour from all other behaviours—unadjusted analysis. For an explanation of this style of graph, please refer to Dumuid *et al.* (2018).

**Figure 2.**
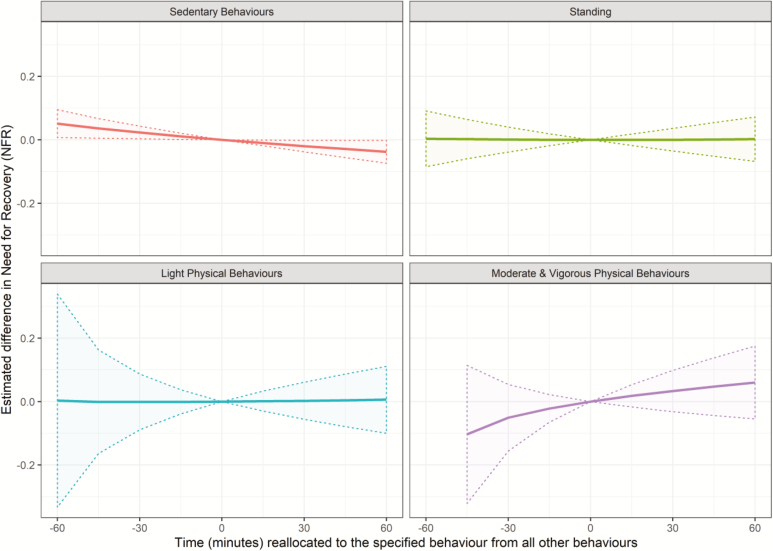
Estimated difference (±95% CI) in NFR (measured on a 5-point Likert scale) when reallocating time to a specific behaviour from all other behaviours—adjusted analysis. For an explanation of this style of graph, please refer to Dumuid *et al.* (2018).

Results of the isotemporal substitution in unadjusted and adjusted models showed significant, but small reductions in NFR when reallocating 30 min to sedentary behaviours from all other behaviours (ΔNFR = −0.025 [−0.041; −0.009] and −0.020 [−0.038; −0.002], respectively; Table 4). Also, despite a lack of significant differences in other reallocations, there was a trend towards increased time spent in more vigorous behaviours being associated with an increased NFR (Table 4 and Figs. 1 and 2). There were no significant effects of reallocating physical behaviours in the one-to-one analyses (Appendix 2, available at *Annals of Work Exposures and Health* online).

**Table 4.** Estimated difference in NFR amongst Danish workers from cleaning, manufacturing, and transportation sectors when reallocating 30 min to the specified behaviour from all other behaviours during working hours.

	Estimated change in NFR [95% CI]	
	Unadjusted model (*N* = 840)	Adjusted model^a^ (*N* = 747)
Sedentary behaviours	**−0.025** **[−0.041; −0.009]**	**−0.020** **[−0.038; −0.002]**
Standing	−0.015 [−0.045; 0.015]	0.000 [−0.035; 0.036]
Light physical behaviours	0.029 [−0.024; 0.082]	0.002 [−0.056; 0.061]
Moderate/vigorous physical behaviours	0.041 [−0.019; 0.100]	0.033 [−0.032; 0.098]

NFR was measured on a 5-point Likert scale, positive values indicate increased NFR; significant values have bolded.

^*a*^Adjusted for age, sex, sector/occupation, and shift work.

**Figure 3.**
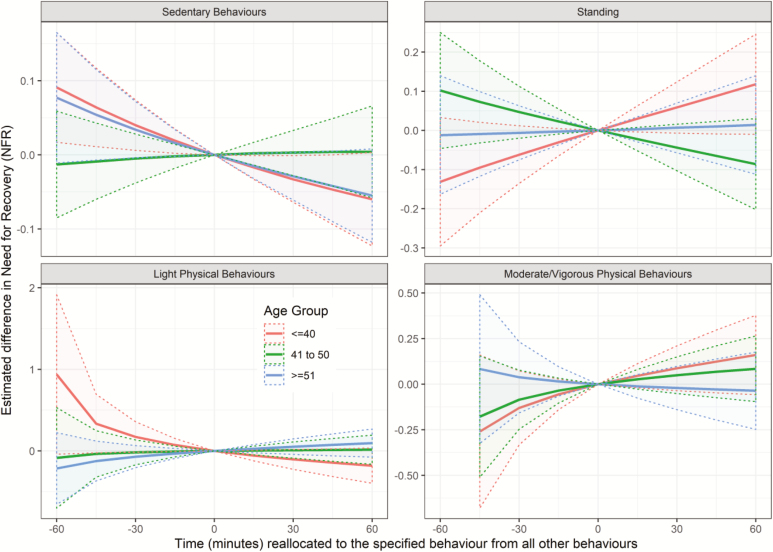
Estimated difference (±95% CI) in NFR (measured on a 5-point Likert scale) when reallocating time to the specified behaviour from all other behaviours—stratified by age. For an explanation of this style of graph, please refer to Dumuid *et al.* (2018).

When stratified by age, the substitution modelling identified significant reductions in NFR when reallocating time to sedentary behaviours from all other behaviours (ΔNFR = −0.033 [−0.064; −0.001]) in those at or below 40 years of age (Table 5 and Fig. 3). When comparing between age groups there were two statistically significant differences. First, increased time standing was associated with an increased NFR in individuals aged 40 or younger but a decreased NFR in those aged 41–50 (*P* = 0.022). Second, increased light physical behaviours were associated with a decreased NFR in those aged 40 or younger but an increased NFR in those aged 51 and older (*P* = 0.046). There were no significant differences between age groups in the one-to-one analyses (Appendix 2, available at *Annals of Work Exposures and Health* online).

**Table 5.** Estimated difference in NFR amongst Danish workers from cleaning, manufacturing, and transportation sectors when reallocating 30 min to the specified behaviour from all other behaviours during working hours—stratified by age.

	Estimated change in NFR [95% CI]		
Age group (years)	≤40 (*n* = 231)	41–50 (*n* = 273)	≥51 (*n* = 243)
Sedentary behaviours	**−0.033** **[−0.064; −0.001]**	0.003 [−0.028; 0.033]	−0.029 [−0.061; 0.003]
Standing	0.059^a^ [−0.007; 0.124]	−0.044^a^ [−0.103; 0.016]	0.007 [−0.057; 0.070]
Light physical behaviours	−0.104^b^ [−0.225; 0.016]	0.009 [−0.090; 0.109]	0.052^b^ [−0.043; 0.147]
Moderate/vigorous physical behaviours	0.087 [−0.036; 0.210]	0.049 [−0.052; 0.150]	−0.021 [−0.140; 0.098]

NFR was measured on a 5-point Likert scale, positive values indicate increased NFR; significant predicted effects on NFR have bolded.

^*a*/*b*^Significant differences between age groups.

### Sensitivity analyses (occupation-stratified analyses)

When stratified by occupation (retaining age, gender, and shift work as potential confounders), the association between the overall behaviour composition and NFR was statistically significant in cleaners (*P* = 0.038) and manufacturing workers (*P* = 0.017) but not in administration workers (*P* = 0.205) or transportation workers (*P* = 0.299). The *β*-coefficients for ilr_1_ showed that, within manufacturing workers, increased total time at work was associated with a significant decrease in NFR (*β* = −0.29; *P* = 0.039). Results for all ilrs are provided in Appendix 1, available at *Annals of Work Exposures and Health* online.

In the occupation-stratified substitution analysis, reallocating time to sedentary behaviours relative to all other behaviours was associated with a significant reduction in NFR in administration workers (ΔNFR = −0.051 [−0.095; −0.007]) and reallocating time to light behaviours relative to moderate/vigorous behaviours was associated with a significant reduction in NFR in cleaners (ΔNFR = −0.281 [−0.545; −0.018]). When considering the differences in associations between occupational groups in the one-to-many analysis, there were significant differences between cleaners and manufacturing workers when reallocating time to standing from all other behaviours at work (*P* = 0.035) and light physical behaviours from all other behaviours at work (*P* = 0.005). The effects for these differences were in opposite directions with increased standing time being associated with an increase in NFR in cleaners and a reduction in NFR in manufacturing workers; whilst increased light behaviours were associated with a reduction in NFR in cleaners and an increase in NFR amongst manufacturing workers. There was also a significant difference between cleaners and transportation workers when reallocating time to moderate/vigorous behaviours from all other behaviours at work (*P* = 0.037). Increased moderate/vigorous behaviours were associated with an increased NFR in cleaners, but a decreased NFR in transportation workers. When considering the differences in associations between occupational groups in the one-to-one analysis, there were significant differences: between cleaners and transportation workers when reallocating time to sedentary behaviours from moderate/vigorous physical behaviours (*P* = 0.016)—with increased sedentary time being associated with a decreased NFR in cleaners but an increased NFR in transportation workers; and between cleaners and manufacturing workers when reallocating time to light physical behaviours from moderate/vigorous physical behaviours (*P* = 0.049)—with this reallocation being associated with a decreased NFR in cleaners but an increased NFR in manufacturing workers. Full details for the occupation-stratified, compositional isotemporal substitution analysis are provided in Appendix 3, available at *Annals of Work Exposures and Health* online.

## Discussion

### Summary of findings

We found a positive association between technically measured OPA and NFR, with a trend towards increased time spent in more vigorous behaviours being associated with increased NFR. However, the effect size was very small, and the only significant reallocation—increasing sedentary time at work relative to all other behaviours—produced only a slight reduction in NFR. There were no significant interactions between age and the overall OPA composition. However, the age stratified results showed significant differences between those aged ≤40 and those aged 41–50 when reallocating time to standing from all other behaviours, and between those ≤40 and those aged ≥51 when reallocating time to light physical behaviours from all other behaviours.

### Strengths and limitations of the study

The key strengths of this study are the relatively large sample size with accelerometer-measured OPA, the use of CoDA to account for the codependency between physical behaviours and the generalizability of our results to multiple occupational groups. However, although the use of accelerometer measures means that the assessment of behaviour is highly accurate, it does not measure important aspects of that behaviour, such as whether the individual is lifting a heavy object. Therefore, the interpretation of this study is limited to the behaviours/postures measured and is not applicable to other related aspects of physical work, such as the intensity of manual handling. The primary limitations of the study are its cross-sectional nature—meaning that the substitution effects are only associations and should not be inferred as necessarily causal—and the relatively short duration of the accelerometer measurement. There may also be limitations associated with the use of a self-reported measure of NFR. As such, physiological measures of NFR may be important in future studies.

### Comparisons with other studies

As this was the first study to use technically measured physical behaviours at work in investigating the association to NFR, and also the first study to use CoDA to account for the codependency of time spent on different behaviours, care must be taken when making comparisons between this study and previous studies, which have measured physical activity indirectly through self-reported measures of physical work demands and workload (Kraaijeveld *et al.*, 2014; Gommans *et al.*, 2016). Nevertheless, although the effect sizes are much smaller in our study, our overall findings generally agree with the results of previous studies that decreasing physical work demands reduces NFR (Kraaijeveld *et al.*, 2014; Gommans *et al.*, 2016). The primary exception to this was a study amongst office workers that showed increased time in physical activities (walking/stair climbing) decreased NFR (Coffeng *et al.*, 2015). This finding is at odds with the results of our stratified occupation-stratified analysis which showed that increasing sedentary time reduced NFR amongst administration workers. This suggests differences between the population studied by Coffeng *et al.* and our own. In particular, Coffeng *et al.* note that this increased activity signified an increase in breaks from work rather than an increase in workload. We do not know if the same holds true for our study.

When considering the effect of age on NFR and its potential to modify the relationship between physical behaviours performed at work and NFR the literature—as with our study—provides no clear answers. Although several studies have noted the modifying effect of age (Kiss *et al.*, 2008; Verdonk *et al.*, 2010; Kraaijeveld *et al.*, 2014; Zacher and Schmitt, 2016), other studies have not (Mohren *et al.*, 2010; Zacher, 2015; Haluza and Blasche, 2016). To explain this, a number of studies have proposed, and found evidence of, healthy-worker effects (Mohren *et al.*, 2010; Gommans *et al.*, 2015; Zacher, 2015), which may also occur in our study.

### Meaning of the study and implications for practitioners and policy makers

The results of this study suggest that decreased time spent in more vigorous behaviours at work is associated with reduced NFR. In particular, increasing sedentary time at work relative to other behaviours was associated with a reduced NFR. However, although there was a significant relationship between increased sedentary time at work and decreased NFR, the size of this relationship was small. This suggests that although the relationship is there, it may not be a useful target for policy makers seeking to reduce NFR amongst workers. As studies generally show associations between physical workload and NFR (Kraaijeveld *et al.*, 2014; Gommans *et al.*, 2016), it may be that other factors associated with workload, apart from the actual physical behaviours performed, might be better targets. For example, although we generally assume that certain physical behaviours link to specific levels of exertion (e.g. little/no exertion when sedentary) this assumption may not be accurate. For instance, an individual that is lifting and carrying heavy objects will be likely to perform a light physical behaviour (slow walking) however will have a high level of exertion. Therefore, it is possible that exertion would be a better target.

In this study, we identified no significant interactions between age and OPA, but the stratified analyses indicated significant differences between age groups in some areas. This is suggestive of two possible explanations. Firstly, the interaction between age and OPA may exist, but has not been observed in this study. If this is the case, it is likely due to bias caused by a healthy-worker effect where older (less able) workers either leave the workforce or move to less demanding roles. Some indications of this in our data are the similar levels of NFR, workability and perceived health across all age groups. The other possible explanation is that no interaction between age and OPA exists. In this case, the significant differences between ages identified in the stratified analysis may be due to the product of multiple hypothesis testing and thus disregarded. Regardless, the marginal effect sizes suggest that these results may not be of practical relevance. Further research is needed to clarify this issue.

The occupation-stratified results show significant differences between the different occupational groupings. Such differences suggest it may be best to analyse these groups separately rather than combine them as we have done. These differences could be due to differences in the time spent conducting different physical behaviours at work (as shown in Table S3-1, available at *Annals of Work Exposures and Health* online), differences between what is actually being conducted within those behaviours (e.g. carrying heavy objects or not) or something else entirely (e.g. work organizational factors). However, similar to the age stratified analysis, the *post hoc* nature of this analysis means that further research is needed to clarify these differences. Moreover, all significant differences between occupational groups involved either cleaners and/or transportation workers—which had small sample sizes—further increasing the likelihood of a type-2 error.

### Future research

Further research in this area needs to address the problems noted above. Firstly, even larger cohorts with homogeneous work-tasks are required to account for the potentially differing effects of physical behaviours across work groups with vastly different work-tasks. Larger samples would also allow for greater age stratification and reduce the uncertainty surrounding our estimates, facilitating the emergence of clear patterns of effect. Secondly, cohorts that collect repeated measures of required outcomes across decades are required to truly remove any systemic bias due to the healthy-worker effect. Moreover, such analyses require complex mediation/moderated-mediation models to assess the impact of age on NFR and understand the various pathways that it acts through. Finally, further research should investigate other factors outside that of the physical behaviours performed, particularly for technically measured physical factors, of which there is very little evidence.

## Conclusions

This first study investigating the associations between accelerometer-measured OPA and NFR suggest that they are related, but the size of these associations was small. Reallocating time to sedentary behaviours from other physical behaviours at work seems to reduce NFR, but the size of this reduction may not be practically relevant. Moreover, no clear modifying effects of age were identified in this study. Further research of the importance of accelerometer-measured OPA on NFR, and of the potential modifying effect age on the relationship between OPA and NFR is required before clear recommendations can be made.

## Supplementary Material

wxz095_suppl_Supplementary_Appendix1

wxz095_suppl_Supplementary_Appendix2

wxz095_suppl_Supplementary_Appendix3
