# Supplementary material for: Accelerometer-Measured Physical Activity at Work and Need for Recovery: A Compositional Analysis of Cross-sectional Data
Source: Ann Work Expo Health. 2019 Dec 27;64(2):138–51. doi: 10.1093/annweh/wxz095 (PMC7031076; doi:10.1093/annweh/wxz095)
Supplement: wxz095_suppl_Supplementary_Appendix1 [file wxz095_suppl_supplementary_appendix1.pdf]

Accelerometer-measured Physical Activity at Work and Need for Recovery: A compositional analysis of cross-sectional data

Matthew L Stevens, Patrick Crowley, Charlotte L Rasmussen, David M Hallman, Ole S Mortensen, Clas-Håkan Nygård, Andreas Holtermann

Appendix 1: isometric log-ratio equations and β-coefficients

Equations

The ilr-coordinates for the 5-part time-use composition were computed as:

$$ilr_1 = \sqrt{\frac{4}{5}} \ln\left(\frac{\sqrt[4]{SB_{work_i} * stand_{work_i} * LMB_{work_i} * MVMB_{work_i}}}{time_{leisure_i}}\right)$$

$$ilr_2 = \sqrt{\frac{3}{4}} \ln\left(\frac{\sqrt[3]{stand_{work_i} * LMB_{work_i} * MVMB_{work_i}}}{SB_{work_i}}\right)$$

$$ilr_3 = \sqrt{\frac{2}{3}} \ln\left(\frac{\sqrt{LMB_{work_i} * MVMB_{work_i}}}{stand_{work_i}}\right)$$

$$ilr_4 = \sqrt{\frac{1}{2}} \ln\left(\frac{MVMB_{work_i}}{LMB_{work_i}}\right)$$

The generic compositional linear regression model was then defined as:

$$y = \beta_0 + \beta_1 ilr_1 + \beta_2 ilr_2 + \beta_3 ilr_3 + \beta_4 ilr_4 + covariates + \varepsilon$$

Table S1-1. β-coefficients and p-values for each ilr for each model run

	ilr_1		ilr_2		ilr_3		ilr_4	
	**β [95%CI]**	**p**	**β [95%CI]**	**p**	**β [95%CI]**	**p**	**β [95%CI]**	**p**
Unadjusted	-0.12 [-0.30; 0.07]	0.214	**0.11 [0.05; 0.17]**	**<0.001**	**0.11 [0.01; 0.21]**	**0.034**	0.01 [-0.14; 0.17]	0.862
Adjusted	-0.05 [-0.24; 0.14]	0.615	**0.08 [0.01; 0.15]**	**0.024**	0.03 [-0.09; 0.16]	0.602	0.04 [-0.12; 0.20]	0.611
**Age stratified**								
Age<=40	-0.21 [-0.52; 0.10]	0.180	**0.16 [0.03; 0.28]**	**0.013**	-0.14 [-0.37; 0.09]	0.238	0.27 [-0.04; 0.58]	0.091
Age 41 to 50	0.09 [-0.25; 0.42]	0.614	-0.02 [-0.14; 0.09]	0.681	0.15 [-0.06; 0.35]	0.173	0.05 [-0.21; 0.31]	0.699
Age>=51	-0.06 [-0.44; 0.33]	0.773	0.11 [-0.02; 0.23]	0.086	0.02 [-0.20; 0.24]	0.848	-0.11 [-0.39; 0.18]	0.453
**Occupation stratified**								
Administration	0.35 [-0.08; 0.79]	0.114	0.05 [-0.15; 0.24]	0.640	-0.03 [-0.28; 0.22]	0.802	0.09 [-0.29; 0.48]	0.632
Cleaning	-0.43 [-1.25; 0.39]	0.296	0.34 [-0.02; 0.69]	0.065	-0.47 [-1.07; 0.13]	0.123	0.67 [0.15; 1.19]	0.012
Manufacturing	**-0.29 [-0.56; -0.01]**	**0.039**	0.07 [-0.01; 0.15]	0.079	0.17 [0.02; 0.33]	0.030	-0.04 [-0.24; 0.15]	0.667
Transportation	-0.10 [-0.69; 0.48]	0.718	-0.07 [-0.49; 0.35]	0.731	-0.37 [-0.93; 0.20]	0.200	-0.36 [-1.29; 0.57]	0.439

The age and occupation stratified models were adjusted models that included sex, shift-work and either sector/occupation or age accordingly.
