# Supplementary material for: Accelerometer-Measured Physical Activity at Work and Need for Recovery: A Compositional Analysis of Cross-sectional Data
Source: Ann Work Expo Health. 2019 Dec 27;64(2):138–51. doi: 10.1093/annweh/wxz095 (PMC7031076; doi:10.1093/annweh/wxz095)
Supplement: wxz095_suppl_Supplementary_Appendix2 [file wxz095_suppl_supplementary_appendix2.pdf]

Accelerometer-measured Physical Activity at Work and Need for Recovery: A compositional analysis of cross-sectional data

Matthew L Stevens, Patrick Crowley, Charlotte L Rasmussen, David M Hallman, Ole S Mortensen, Clas-Håkan Nygård, Andreas Holtermann

Appendix 2 – One-to-One Analyses

Table 1. Estimated difference in Need for Recovery (NFR) among Danish workers from cleaning, manufacturing and transportation sectors when reallocating 30 minutes from Moderate/Vigorous Movement Behaviours to the specified behaviour during working hours

	**Estimated change in NFR [95%CI]**	
	**Unadjusted Model** (n=840)	**Adjusted Model** [a] (n=747)
**Sedentary Behaviours**	-0.068 [-0.161; 0.025]	-0.058 [-0.158; 0.042]
**Standing**	-0.063 [-0.151; 0.026]	-0.046 [-0.146; 0.055]
**Light Movement Behaviours**	-0.030 [-0.152; 0.092]	-0.044 [-0.175; 0.087]

NFR was measured on a 5 point Likert scale, positive values indicate increased NFR
[a] adjusted for age, sex, sector/occupation and shift-work

Table 2. Estimated difference in Need for Recovery (NFR) among Danish workers from cleaning, manufacturing and transportation sectors when reallocating 30 minutes from Moderate/Vigorous Movement Behaviours to the specified behaviour during working hours – stratified by age

	**Estimated change in NFR [95%CI]**		
**Age Group (years)**	**<=40** (n=231)	**41 to 50** (n=273)	**>=51** (n=243)
**Sedentary Behaviours**	-0.136 [-0.328; 0.056]	-0.075 [-0.230; 0.080]	0.016 [-0.169; 0.202]
**Standing**	-0.079 [-0.271; 0.112]	-0.104 [-0.257; 0.048]	0.039 [-0.150; 0.227]
**Light Movement Behaviours**	-0.202 [-0.461; 0.058]	-0.069 [-0.278; 0.140]	0.075 [-0.157; 0.307]

NFR was measured on a 5 point Likert scale, positive values indicate increased NFR

Figure 1. Estimated difference (±95%CI) in Need for Recovery (measured on a 5 point Likert scale) when reallocating time to Moderate/ Vigorous Physical Behaviours from the specified behaviours – unadjusted analysis

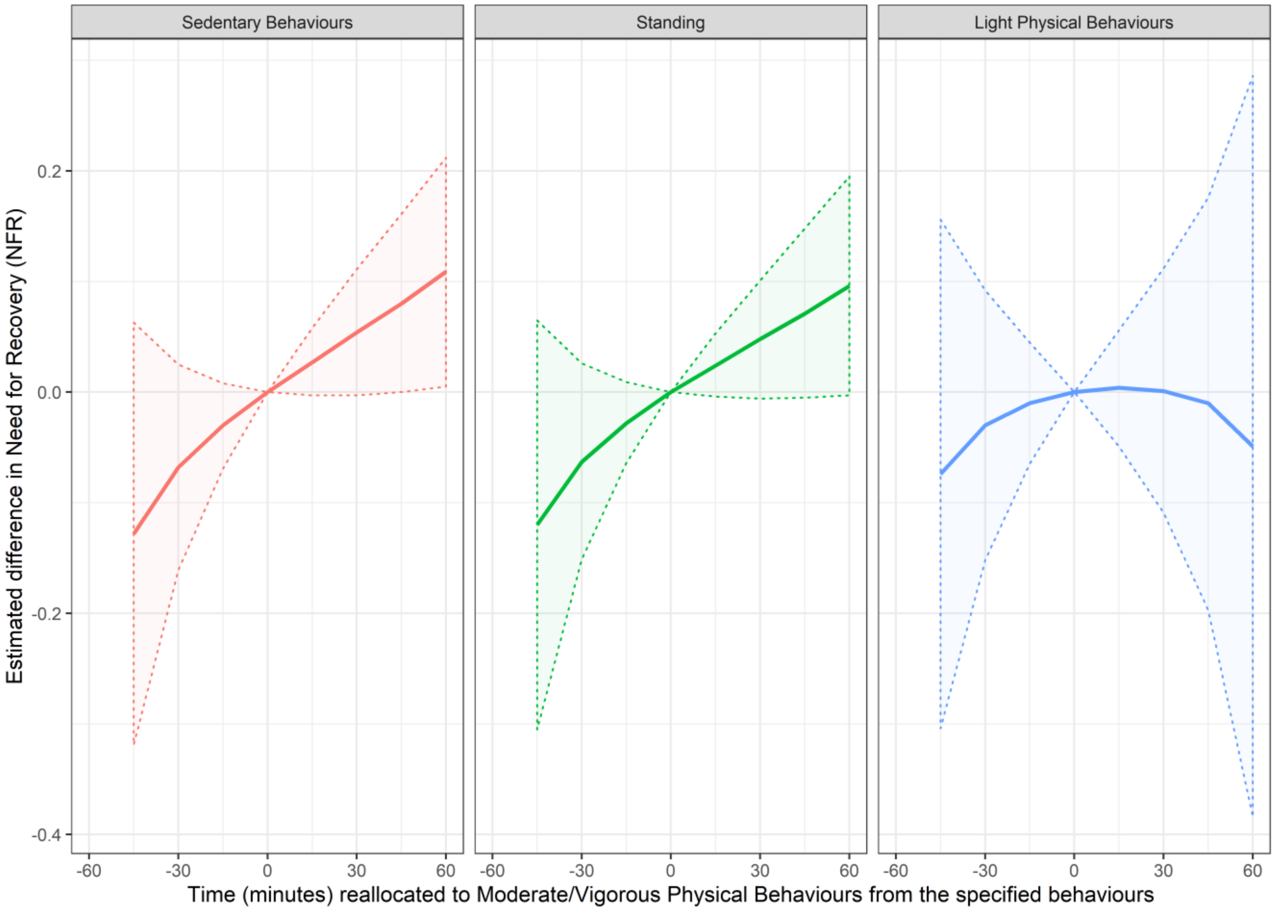

For an explanation of this style of graph, please refer to Dumuid et al., 2018

Figure2. Estimated difference (±95%CI) in Need for Recovery (measured on a 5 point Likert scale) when reallocating time to Moderate/ Vigorous Physical Behaviours from the specified behaviours – adjusted analysis

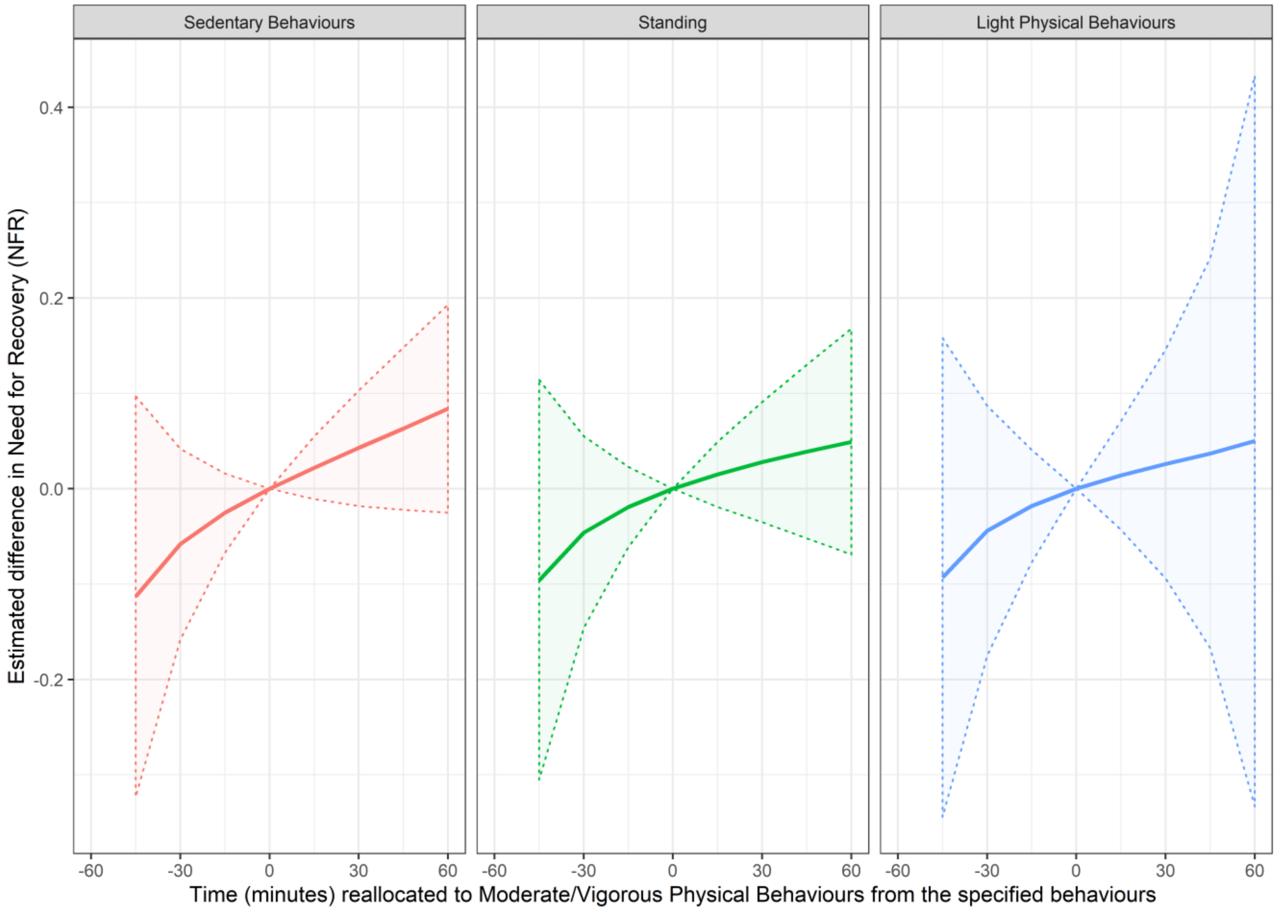

For an explanation of this style of graph, please refer to Dumuid et al. (2018)

Figure 3. Estimated difference (±95%CI) in Need for Recovery (measured on a 5 point Likert scale) when reallocating time to Moderate/Vigorous Physical Behaviours from all other behaviours – stratified by age

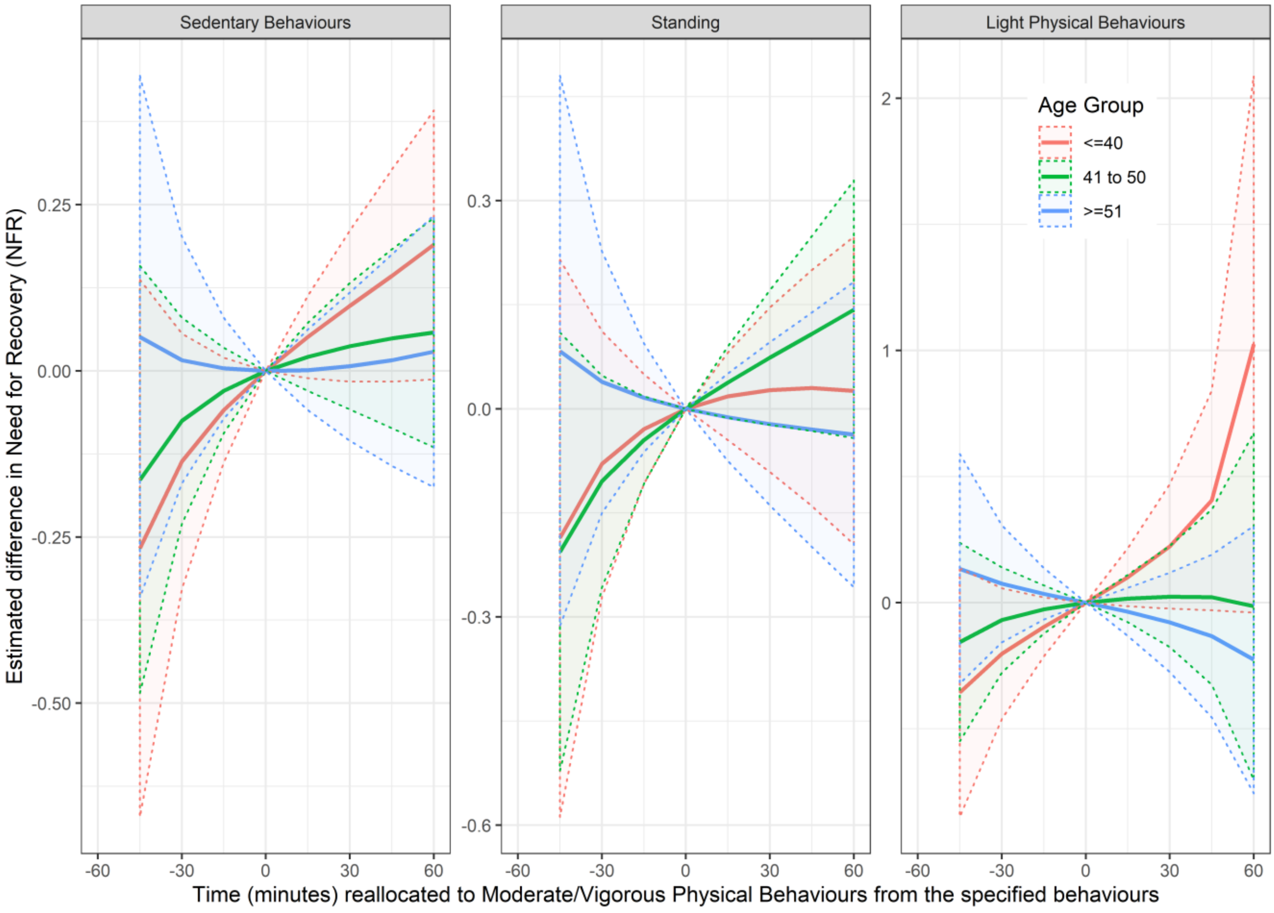

For an explanation of this style of graph, please refer to Dumuid et al. (2018)

Reference

Dumuid D, Stanford, TE, Martin-Fernández J-A, Pedišić Ž, Maher CA, Lewis LK, Hron K, Katzmarzyk, PT, Chaput J-P, Fogelholm M, Hu G, Lambert EV, Maia J, Sarmiento OL, Standage M, Barreira TV, Broyles ST, Tudor-Locke C, Tremblay MS, Olds T. (2018) Compositional data analysis for physical activity, sedentary time and sleep research. Stat Methods Med Res; 27:3726–38.
