# Supplementary material for: Accelerometer-Measured Physical Activity at Work and Need for Recovery: A Compositional Analysis of Cross-sectional Data
Source: Ann Work Expo Health. 2019 Dec 27;64(2):138–51. doi: 10.1093/annweh/wxz095 (PMC7031076; doi:10.1093/annweh/wxz095)
Supplement: wxz095_suppl_Supplementary_Appendix3 [file wxz095_suppl_supplementary_appendix3.pdf]

Accelerometer-measured Physical Activity at Work and Need for Recovery: A compositional analysis of cross-sectional data

Matthew L Stevens, Patrick Crowley, Charlotte L Rasmussen, David M Hallman, Ole S Mortensen, Clas-Håkan Nygård, Andreas Holtermann

Appendix 3 – Stratification by Occupation

Table S3-1. Mean time spent in Physical Behaviour types at work for workers from cleaning, manufacturing and transportation sectors in Denmark

	Geometric Mean (mins/day)				
Occupation Group	SB	Standing	LPB	MVPB	Non-work
Administration [a] (n=181)	257	95	32	38	1018
Blue-collar					
Cleaning (n=79)	84	115	120	80	1040
Manufacturing (n=445)	114	163	80	57	1026
Transportation (n=47)	260	53	45	74	1008

[a] The administration group consisted of administration workers from all three sectors (cleaning, manufacturing and transportation)
SB = Sedentary Behaviours (lying, sitting)
LPB = Light Physical Behaviours (dynamic standing, slow walking)
MVPB = Moderate/Vigorous Physical Behaviours (fast walking, running, stair climbing, cycling)
Non-work = time spent outside work (i.e. leisure, sleep, transportation) on a work day

Table S3-2. Estimated difference in Need for Recovery (NFR) among Danish workers from cleaning, manufacturing and transportation sectors when reallocating 30 minutes to the specified behaviour from all other behaviours during working hours

	**Predicted change in NFR [95%CI]**			
	**Admin** (n=181)	**Blue-collar Workers**		
		**Cleaning** (n=78)	**Manufacturing** (n=442)	**Transportation** (n=46)
**Sedentary Behaviours**	**-0.051** **[-0.095; -0.007]**	-0.118 [-0.244; 0.007]	-0.016 [-0.038; 0.007]	0.030 [-0.070; 0.130]
**Standing**	0.013 [-0.068; 0.095]	0.174 [a] [-0.004; 0.353]	-0.023 [a] [-0.063; 0.018]	0.143 [-0.127; 0.414]
**Light Physical Behaviours**	-0.017 [-0.233; 0.200]	-0.180 [a] [-0.325; 0.035]	0.048 [a] [-0.014; 0.110]	0.044 [-0.410; 0.499]
**Moderate/Vigorous Physical behaviours**	0.070 [-0.124; 0.264]	0.157 [a] [-0.032; 0.345]	0.021 [-0.056; 0.099]	-0.184 [a] [-0.448; 0.081]

NFR was measured on a 5 point Likert scale; significant values have bolded; analyses have been adjusted for age, sex, sector/occupation and shift-work
[a] significant difference between specified occupational groups (p<0.05)

Table S3-3. Estimated difference in Need for Recovery (NFR) among Danish blue-collar workers from cleaning, manufacturing and transportation sectors when reallocating 30 minutes from Moderate/Vigorous Physical Behaviours to the specified behaviour during working hours

	**Predicted change in NFR [95%CI]**			
	**Admin** (n=181)	**Blue-Collar workers**		
		**Cleaning** (n=78)	**Manufacturing** (n=442)	**Transportation** (n=46)
**Sedentary Behaviours**	-0.217 [-0.671; 0.238]	-0.251 [a] [-0.509; 0.007]	-0.028 [-0.148; 0.093]	0.238 [a] [-0.090; 0.566]
**Standing**	-0.189 [-0.644; 0.267]	-0.044 [-0.326; 0.237]	-0.030 [-0.148; 0.088]	0.348 [-0.075; 0.771]
**Light Physical Behaviours**	-0.213 [-0.820; 0.394]	**-0.281** [a] **[-0.545; -0.018]**	0.020 [a] [-0.127; 0.168]	0.265 [-0.420; 0.951]

NFR was measured on a 5 point Likert scale; significant values have bolded; analyses have been adjusted for age, sex, sector/occupation and shift-work

[a] significant difference between specified occupational groups ($p<0.05$)

Figure S3-1. Estimated difference (±95%CI) in Need for Recovery (measured on a 5 point Likert scale) among Danish workers from cleaning, manufacturing and transportation sectors when reallocating time to a specific behaviour from all other behaviours – adjusted analysis

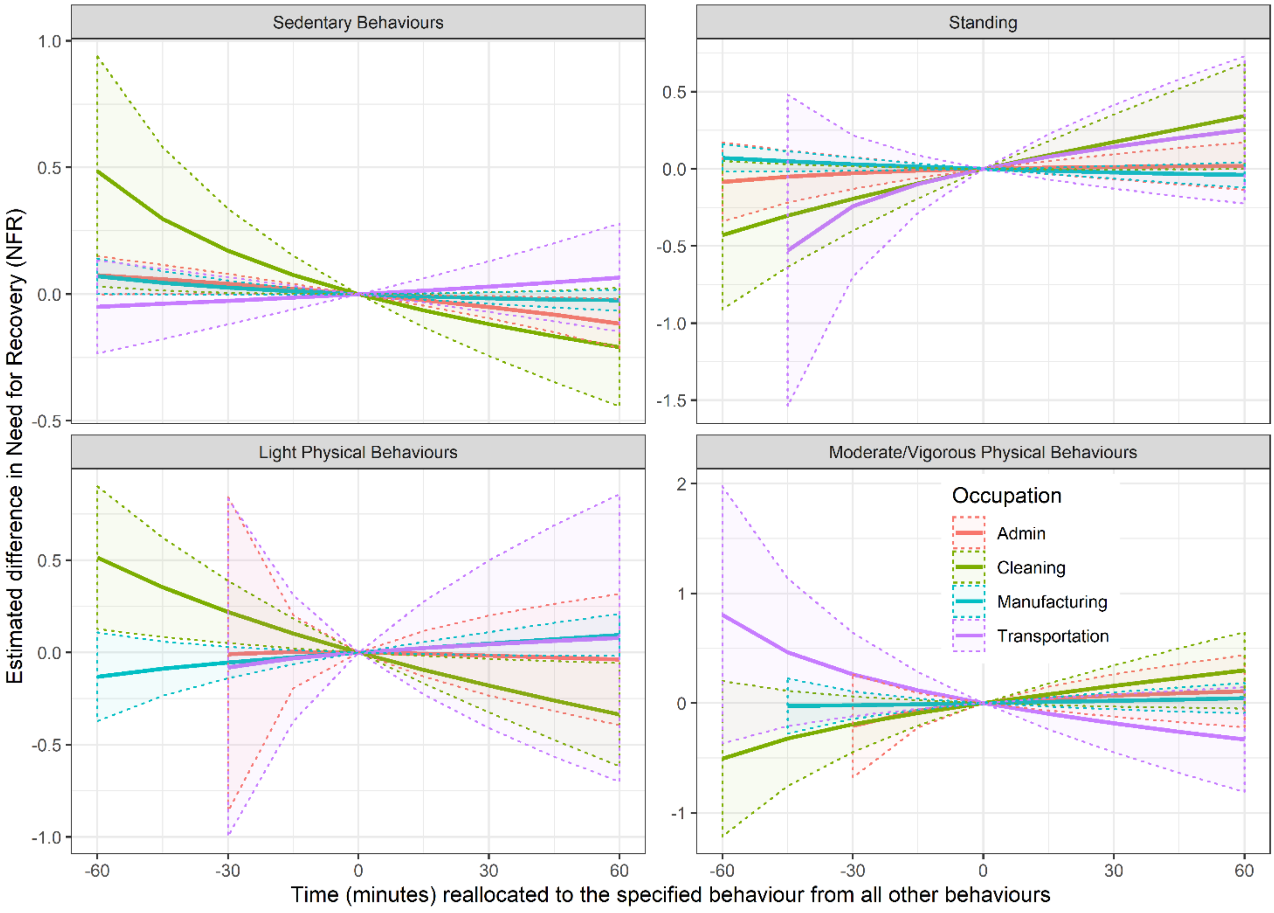

For an explanation of this style of graph, please refer to Dumuid et al. (2018)

Figure S3-2. Estimated difference (±95%CI) in Need for Recovery (measured on a 5 point Likert scale) among Danish workers from cleaning, manufacturing and transportation sectors when reallocating time to Moderate/ Vigorous Physical Behaviours from the specified behaviours – adjusted analysis

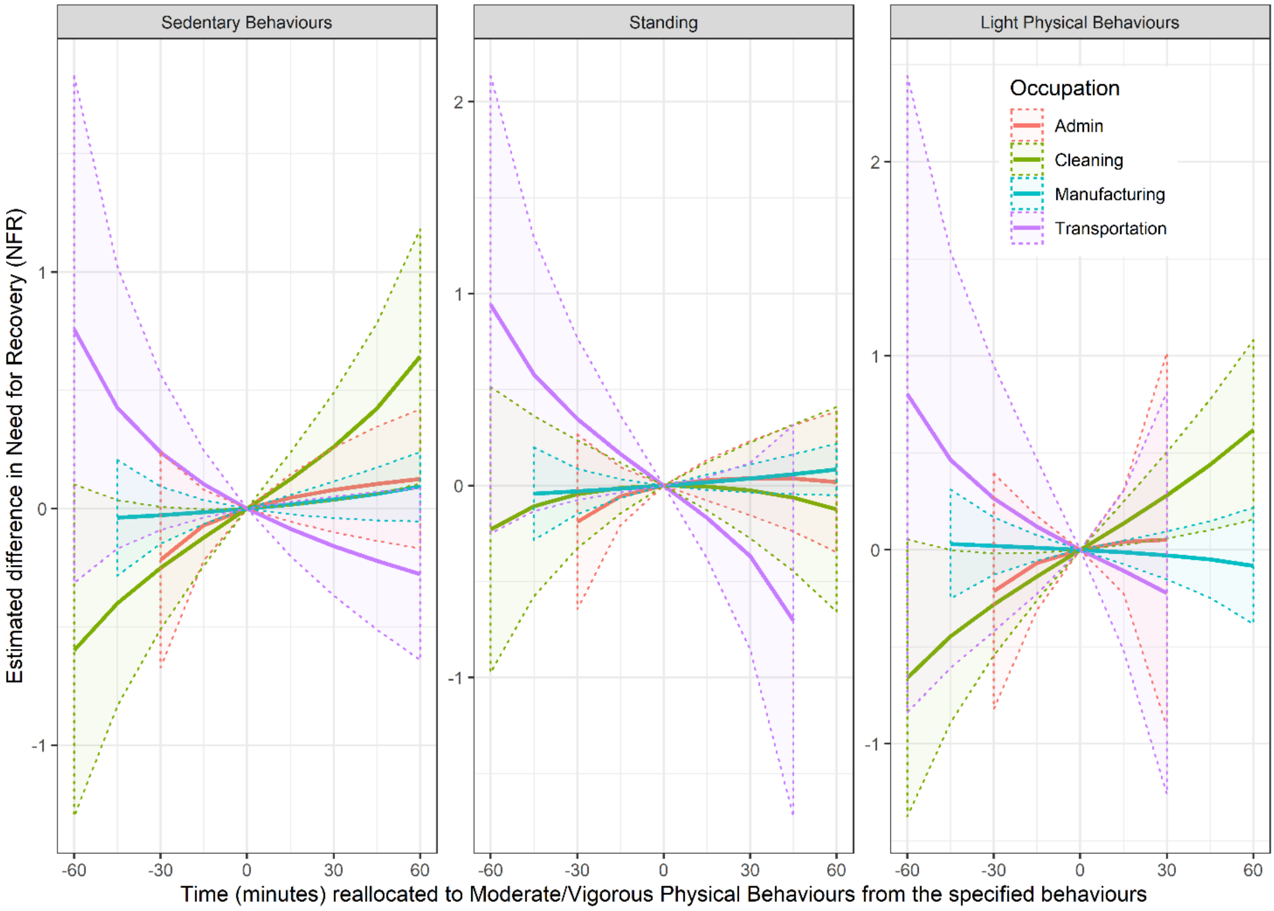

For an explanation of this style of graph, please refer to Dumuid et al. (2018)

Reference

Dumuid D, Stanford, TE, Martin-Fernández J-A, Pedišić Ž, Maher CA, Lewis LK, Hron K, Katzmarzyk, PT, Chaput J-P, Fogelholm M, Hu G, Lambert EV, Maia J, Sarmiento OL, Standage M, Barreira TV, Broyles ST, Tudor-Locke C, Tremblay MS, Olds T. (2018) Compositional data analysis for physical activity, sedentary time and sleep research. Stat Methods Med Res; 27:3726–38.
